# Vesiculobullous disease with Cutaneous Systemic Lupus Erythematosus Treated by Rituximab: A case report

**DOI:** 10.1002/ccr3.4967

**Published:** 2021-10-15

**Authors:** Wigdan Mohammed Niamat Alla, Burhan Mohamed Ali Ahmed, Mohammed Elmujtba Adam Essa, Atif Elhadi Abdalla Babker, Elnour Mohammed Elagib

**Affiliations:** ^1^ Department of Rheumatology Sudan Medical Specialization Board Khartoum Sudan; ^2^ Department of Clinical Medicine Medical and Cancer Research Institute Khartoum Sudan; ^3^ Faculty of Medicine Alfashir University Alfashir Sudan; ^4^ Faculty of Medicine University Of Gezira wad Medani Sudan; ^5^ Department of Internal Medicine and Rheumatology Omdurman Military hospital Khartoum Sudan

**Keywords:** oral ulceration, rituximab, systemic lupus erythematosus, vesiculobullous

## Abstract

Vesiculobullous lesions in systemic lupus erythematosus (SLE) are a rare cutaneous manifestation of cutaneous and/or systemic LE with variable presentation. The diagnosis of SLE‐associated vesiculobullous diseases remains challenging, due to the poorly defined similarities and nosology in immunohistopathological and clinical and features.

## Graphical abstract

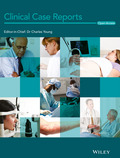

## 1 INTRODUCTION

Systemic lupus erythematosus (SLE) is a chronic autoimmune disease characterized by multisystem inflammation, vesiculobullous (VB) is different groups of oral disorders characterized by the formation of bullae. The aim of this report is to describe a case of a VB in SLE. A 43‐year‐old Sudanese housewife presented to our unit with recurrent painless oral ulceration associated with joints pain, morning stiffness, and generalized fatigue. She was diagnosed with SLE a year ago, and for which she is taking prednisolone, hydroxychloroquine (HCQ), and azathioprine. She also developed left side weakness of sudden onset. Immunological and radiological investigations showed a diagnosis of VB. The patient received hydrocortisone, pantoprazole, paracetamol, mebo cream, fusiderm cream, B.protien powder, metronidazole, ceftriaxone, flucloxacillin and Tonics, and IV Immunoglobulin (IVIM). Although her condition was not well improved. A week later rituximab has been administrated and the patient showed a dramatic response to it. Now she is on regular follow‐up. In conclusion, a middle age female diagnosed with SLE presented with joint pain and features suggested a diagnosis of VB, she received the regiment treatment but her condition was refractory hence treated with rituximab.

SLE is an autoimmune condition with a variable range of clinical features from skin disease to systemic involvement.^1^, ^2^ VB diseases are a distinct group of oral disorders characterized by the formation of vesicles or bullae.^3^ Bullous systemic lupus erythematosus (BSLE) is a rare blistering condition that mainly happens in females,^4^ the incidence of SLE is about 1 to 10/100,000 annually, and its prevalence is around 6–130/100,000.^5^ Approximately 60%–85% of SLE patients have cutaneous involvement; however, near 5% may develop vesiculobullous lesions, which occur in the form of bullae, vesicles, erosions, and sometimes crusts.^6^

BSLE is marked by the rapid and widespread progress of tense bullae and vesicles over erythematous macules or plaques. Preferential sites are as follows: proximal superior limbs, superior trunk, and the face. Mucosal involvement is common in the laryngeal, pharyngeal, perioral, and genital areas. The lesions usually develop with no scarring, but hyper or hypochromia can occur.^7^ Here, we believed that this case report will receive the most attention from Sudanese clinicians and rekindle knowledge and awareness in regard to vesiculobullous disease in a patient who is known to be suffering from SLE.

## 2 CASE REPORT

**FIGURE 1:**
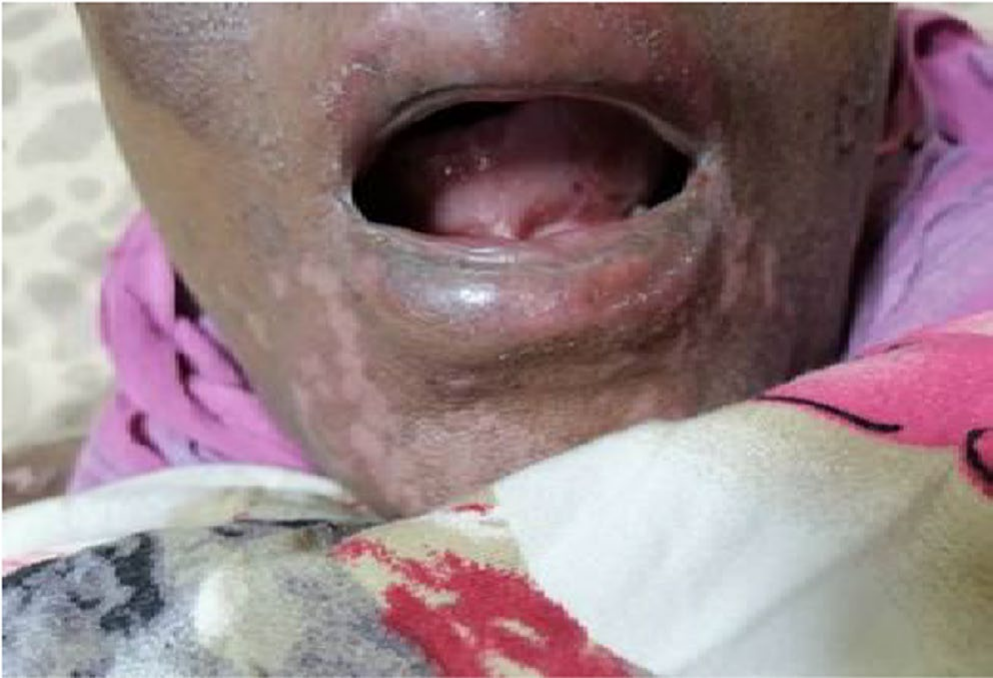
Showed the oral ulcer

**FIGURE 2:**
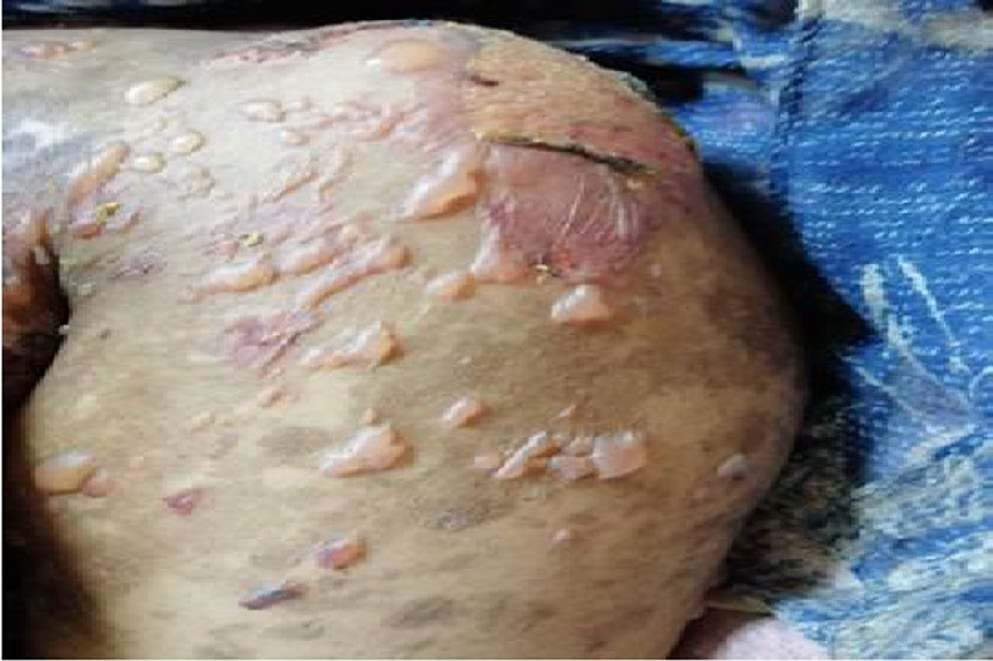
Showed tense bullae over erythematous macules

**FIGURE 3:**
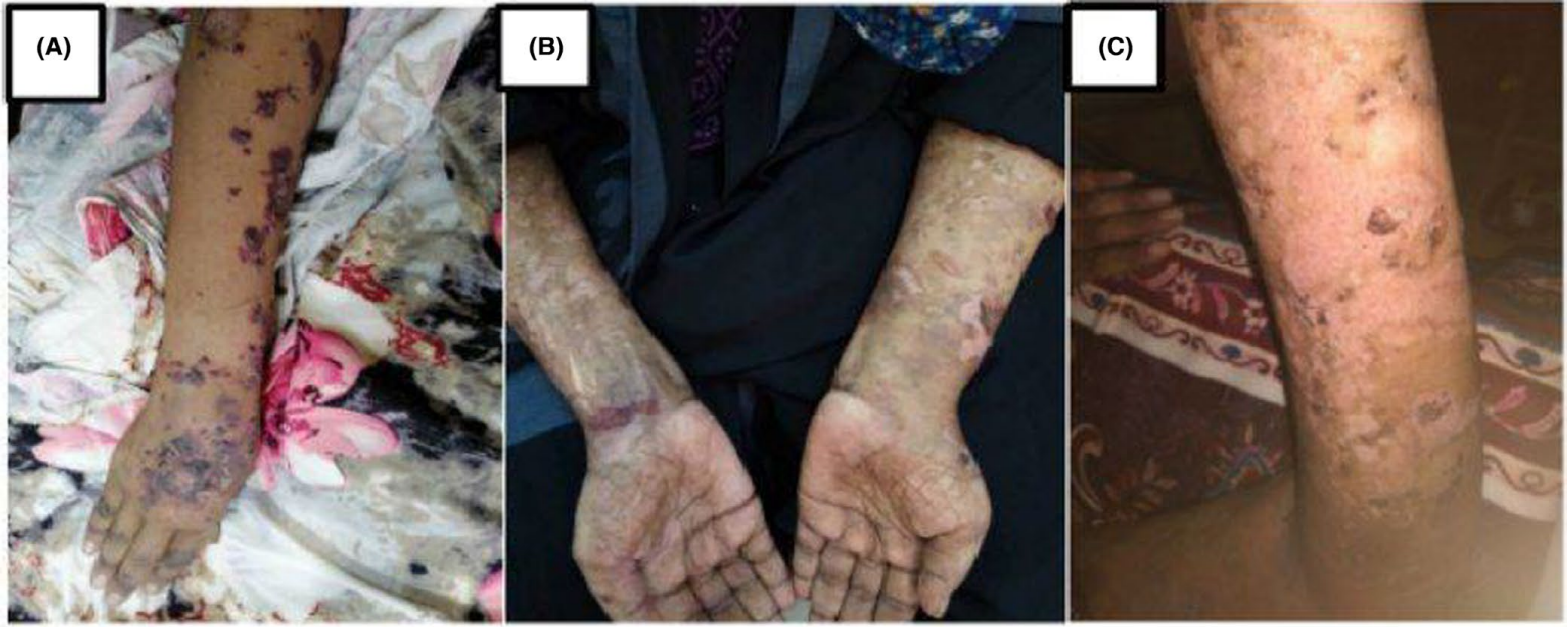
Showed linear tense bullae of varying sizes over erythematous macules and plaques, A: showed the bullae before treatment, B: during treatment, C: after rituximab administration

A 43‐year‐old Sudanese housewife, presented on June 2021 at Omdurman military hospital, Khartoum, Sudan. With recurrent painless oral ulceration (Figure 1) for the last 2 years associated with joints pain affecting the metacarpophalangeal joints (MCP), proximal interphalangeal joints (PIP), wrist joint of both hands, morning stiffness lasting for more than an hour, and generalized fatigue. Although she has no skin rash, genital ulcers, muscle pain, dry eye, or mouth. In September 2020, she was diagnosed with SLE based on her clinical presentation and the positive finding of ANA profile which was strongly positive for Ribonucleoproteins Abs (RNP), anti‐Smith Abs(Sm), anti‐Ribosomal Proteins Abs, (Ribp) and microalbuminuria. For which, Prednisolone 20 mg, hydroxychloroquine (HCQ) 200 mg, and azathioprine 50 mg were prescribed. Two months later, after the oral ulceration minimally improved new lesions in form of macular rash with vesicle and blister start to appear in her mouth, face, trunks, upper and lower limbs (Figure 2). When the blister is ruptured they lifted a hyper and hypopigmentation mark without scarring. During this period, she lost considerable weight and has a fever, hair loss, and headache. The patient has been referred to the dermatology department, in which a blood test for vitamin b12 level and desmoglein 1 and 3 were ordered, and their result was normal; therefore, topical treatments were prescribed without a clear diagnosis. After 5 months, the patient developed left side weakness of sudden onset. Although her cardiovascular, respiratory, gastrointestinal, and genitourinary systems were unremarkable. She has no history of any neurological disease, diabetes, or hypertension. She has a family history of SLE related to her sister. Her clinical examination upon admission showed generalized thin‐walled bullae, some of them are hemorrhagic with erythematous macule and area of hyper and hypopigmentation; however, no evidence of active synovitis was detected. Central nervous system examination showed no sign of meningism or cranial nerve involvement, she was conscious, oriented with Glasgow Coma Scale (GCS) 15/15. The left side of her body revealed an increased tone and reflex, power grade three with an upgoing plantar reflex, and a normal right side. Full workup was done showed low hemoglobin, high erythrocyte sedimentation rate (ESR), and C reactive protein 13, Normal urine analysis without active sediment, renal profile, liver profile, Vitamin B12 level, Antiphospholipid antibodies, chest X‐ray, echocardiography, electrocardiograph, viral screening, and lipid profile were all normal. Antinuclear antibody (ANA) profile showed strong positive (RNP/Sm ab, Sm ab, anti‐ RibP ab), urine for albumin/creatinine ratio was 9.6 mg/mmol (microalbuminuria), Complement 3 (C3) and Complement 4 (C4) were low. Desmoglien1 Abs is 16, desmoglein 3 Abs is 18. Based on the clinical and laboratory investigation, a diagnosis of vesiculobullous disease with SLE was established. The patient received Hydrocortisone intravenously (IV) 100 mg, Pantoprazole 40 mg IV, Paracetamol 1 g iv along with Mebo cream, fusiderm cream and B.protien powder, Metronidazole 500 mg iv, Ceftriaxone 1 gm, flucloxacillin and Tonics, and IVIM 4 mg/KG/day for 3 days. Her condition was improved partially However, bullous formation still continues to appear, hence, rituximab 500 mg/weekly for 4 weeks has been administrated followed by mycophenolate mofetil 500 mg BD. A few days later, the patient discharge with marked response to the treatment (Figure 3). Now she is on regular follow‐up.

## 3 DISCUSSION

A vesiculobullous disease is a rare autoimmune blistering disease that commonly occurs as tense bullae with or without vesicles in patients with SLE.^8^ Due to the rarity of the condition, his epidemiology data are restricted, however, some reports showed an incidence of 3.4/million/year. The incidence of this disease with SLE is about 1%. As the knowledge about it is mainly based on case series or reports.^7^, ^9^ Patients with the vesiculobullous disease usually present with clinical features of SLE, the blisters mainly occur on the oral mucosa, neck, face, and upper extremities.^10^ All the reported cases indicated these bullae were mostly triggered by sun exposure, they were also tense and heal with no scarring.^8^ This is a classical presentation of our reported case as it is already diagnosed with SLE, the bullae occurred on the mouth, face, neck, and extremities. It was tense and was healed without any scars behind.

The pathophysiology of VB is mostly multifactorial, which includes environmental triggers, genetic predisposition, and ultraviolet aggravation leading to adaptive and innate immune response.^11^ Activation of the immune system leads to the activation of self‐antigen‐specific T cells, autoreactive cytotoxic T lymphocytes from dermal autoantibody deposition cause hydropic degeneration of the basal‐cell layer of the epidermis and apoptotic keratinocytes.^12^ The diagnosis of VB diseases should be done according to immunological, histopathological, and clinical grounds. Mucosal disorder and bullae can be diagnosed from clinical examination and brief history. The histopathology commonly shows sub‐epidermal bullae with neutrophil‐predominant inflammatory infiltrate below the bullae formation. Also, microabscesses can be present in the dermal papillae is also frequent.^13^ Immunological assay for VB mainly can be done by detecting high desmoglein antibodies (DSG), DSG2 was found in all desmosome‐possessing tissues, such as nonepithelial myocardium; however, the expression of DSG1 and DSG3 was mainly limited to the epidermis and mucosa.^14^ DSG delivered insight into the pathophysiology of a more common disease, bullous as well as the associated disease.^15^ In our patient, both DSG1 and DSG2 were significant to the diagnosis. The erosions and blisters are formed by circulating immunoglobulin (IgG) autoantibodies against DSG1 and DSG3, adhesion molecules in desmosomes that have an essential role in the cohesion between keratinocytes.^16^ The first treatment of autoimmune bullous diseases is systemic corticosteroids in combination with other immunosuppressants drugs to decrease autoantibody production.^17^ In most patients, this course of treatment is effective; however, in refectory cases, more targeted drugs against autoantibody production such as rituximab are used.^18^

Limitation of the study: In this report, the patient refused to undergo though the tissue biopsy procedure, so the diagnosis was mainly based on immunological assay and clinical ground.

In conclusion, a middle‐aged Sudanese woman with SLE, presented with painless oral ulcer and joint pain for the last 2 years during the treatment she developed vesicles and blisters in most of her body part, clinical and immunological investigation revealed a diagnosis of VB associated with SLE, the patient received regiment treatment of steroids but her condition not improved, then rituximab has been used, which showed good response. Rituximab is a monoclonal antibody against CD 20, has been reported to be effective in refractory VD, is act through deplete antibody‐producing B cells a standard dose of it has been shown to offer long‐term efficacy in patients with refractory VD.^6^ At present, there is no officially approved indication for rituximab in dermatologic conditions, although the drug is included as a treatment option for certain dermatological diseases when conventional treatment has not been effective.^16^

## CONFLICT OF INTEREST

None.

## AUTHOR CONTRIBUTION

All authors contributed equally.

## ETHICS APPROVAL AND CONSENT TO PUBLISH

Obtained.

## CONSENT

Obtained.

## Data Availability

All the data used in the study are available from the first and corresponding author on reasonable request.
